# A *Bos taurus* sequencing methods benchmark for assembly, haplotyping, and variant calling

**DOI:** 10.1038/s41597-023-02249-1

**Published:** 2023-06-08

**Authors:** Camille Eché, Carole Iampietro, Clément Birbes, Andreea Dréau, Claire Kuchly, Arnaud Di Franco, Christophe Klopp, Thomas Faraut, Sarah Djebali, Adrien Castinel, Matthias Zytnicki, Erwan Denis, Mekki Boussaha, Cécile Grohs, Didier Boichard, Christine Gaspin, Denis Milan, Cécile Donnadieu

**Affiliations:** 1 INRAE, US 1426, GeT-PlaGe, Genotoul, France Genomique, Université Fédérale de Toulouse, Castanet-Tolosan, France; 2 Université Fédérale de Toulouse, INRAE, BioinfOmics, GenoToul Bioinformatics facility, 31326 Castanet-Tolosan, France; 3 GenPhySE, Université de Toulouse, INRAE, INPT, ENVT, Castanet-Tolosan, 31326 France; 4 IRSD, Université de Toulouse, INSERM, INRAE, ENVT, UPS, 31024 Toulouse, France; 5 Université Fédérale de Toulouse, INRAE, MIAT, 31326 Castanet-Tolosan, France; 6 Université Paris-Saclay, INRAE, AgroParisTech, GABI, 78350 Jouy-en-Josas, France

**Keywords:** Animal breeding, Genome assembly algorithms, Next-generation sequencing, Structural variation

## Abstract

Inspired by the production of reference data sets in the Genome in a Bottle project, we sequenced one Charolais heifer with different technologies: Illumina paired-end, Oxford Nanopore, Pacific Biosciences (HiFi and CLR), 10X Genomics linked-reads, and Hi-C. In order to generate haplotypic assemblies, we also sequenced both parents with short reads. From these data, we built two haplotyped trio high quality reference genomes and a consensus assembly, using up-to-date software packages. The assemblies obtained using PacBio HiFi reaches a size of 3.2 Gb, which is significantly larger than the 2.7 Gb ARS-UCD1.2 reference. The BUSCO score of the consensus assembly reaches a completeness of 95.8%, among highly conserved mammal genes. We also identified 35,866 structural variants larger than 50 base pairs. This assembly is a contribution to the bovine pangenome for the “Charolais” breed. These datasets will prove to be useful resources enabling the community to gain additional insight on sequencing technologies for applications such as SNP, indel or structural variant calling, and *de novo* assembly.

## Background & Summary

The Charolais breed, which originates from Central France, is the leading suckling cattle breed in Europe, representing 25% of the total cows. Beyond Europe, the population has an international extension. It is particularly developed on grazing and extensive production systems with excellent maternal qualities combined with a high growth potential and an excellent beef conformation. In spite of the economic and social importance of this breed, the specificities of its genome remain poorly known and justify an in-depth characterization effort.

Genome sequencing has evolved at a very fast pace in decades. Whereas only short reads were available less than ten years ago, we now have access to much longer reads, which make it possible to build high quality de novo genome assemblies. Currently, these long reads are produced by two sequencing technologies. The Oxford Nanopore Technology (ONT) sequencers produce very long reads, but are fraught with errors. The Pacific Biosciences (PacBio) sequencers offer two sequencing modes: Continuous Long Read (CLR), which provides long reads with high error rate; and Circular Consensus Sequences (CCS), also called HiFi, which provides reads somewhat shorter and almost devoid of errors.

While both these technologies do improve genome assembly quality, we still observe that some regions are missing or cannot be resolved, due to repetitiveness distance between them, or even fill gaps. Mate-pairs were, until a few years, the most used method to do so. We now have access to more advanced technologies, which include linked reads and Hi-C.

Choosing the best mix of technologies for a desired assembly quality requires a deep knowledge of the field. Moreover, incessant improvements in sequencing technologies usually make previous ones obsolete, and drive users to switch from one mix of technologies to another. In order to help the community, several benchmarks are already available. The type of repeated sequences present in the genome strongly influences the ability to establish a complete genome assembly. There was therefore a great interest in having another type of dataset next to the already available benchmarks such as the human dataset proposed by GIAB.

The SeqOccIn project aims to explore the use of new sequencing technologies in agronomic research. We created reference datasets for genome assembly, haplotyping, and variability discovery for species of agronomic interest. In this article, we describe the datasets corresponding to the sequencing of a Charolais heifer. In order to infer her haplotypes, we also sequenced her parents.

**Fig. 1:**
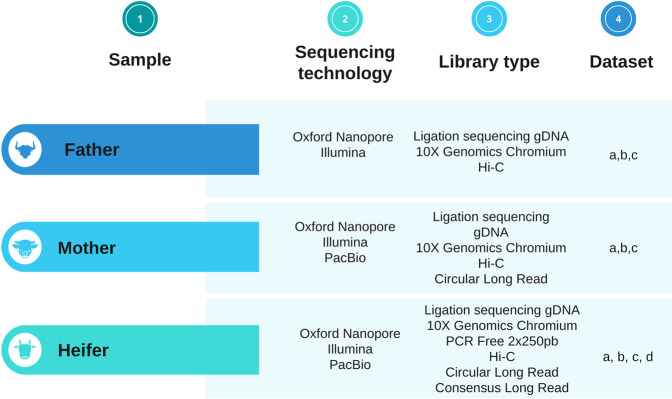
Technologies used for the study. The parents and the heifer were sequenced with Oxford Nanopore Technologies on GridION and PromethION, Chromium 10X and the Hi-C method on MiSeq, HiSeq or NovaSeq 6000. The heifer was additionally sequenced with Illumina 2 × 250 bp on NovaSeq 6000 and PacBio Sequel II (CLR and CCS (i.e HiFi reads) mode). For the Trio approach, parent reads (2 × 150bp) from 10X Genomics Chromium datas were used.

We produced 13 datasets using 3 sequencing technologies and 6 library preparation methods (see Fig. 1). These datasets include high-depth paired-end short read whole genome sequencing (WGS), Hi-C, linked reads WGS, long read WGS, as well as metadata. For each dataset, we describe the library preparation and sequencing methods, the currently available data records, and technical validation.

Thanks to these datasets, we were able to provide a high quality assembly of a bovine Charolais heifer. A haplotype-resolved as well as a consensus assembly were produced. The size of our best assembly, using Pacific Biosciences HiFi, is 3.2 Gb, compared to 2.7 Gb for the reference assembly, ARS-UCD1.2; our assembly contains 1444 contigs and has an N50 of 87 Mb, compared to 2596 contigs and an N50 of 25 Mb for the reference assembly (see Table 3). After scaffolding using Hi-C data, our assembly reaches an N50 of 88 Mb, with 1391 scaffolds (see Table 4). The 29 autosomes and the X chromosome are covered by 1.7 scaffolds on average. In addition, we used Illumina reads from the parents in order to produce phased assemblies with similar metrics: an N50 of 70 Mb, and 3.15 Gb total size with less than 3% missphased k-mers. With the current state of sequencing technologies, the approach we currently recommend is a combination of short fragment sequencing on both parents with PacBio Hifi sequencing of the progeny to be assembled, allowing the separation of reads from each parental chromosome set. Although made from reads of a limited size of 12–15 kb, the very low sequencing error rate allows the inclusion of most of the repeated areas in the assembly thanks to the few SNPs making the repeated sequences present in the genome almost unique. Parent short reads data allowing identification of the correct parental origin of independent contigs.

## Methods

### Genome information

Domestic cattle genome follows the *Bovidae* family chromosomal organization with 29 autosomes and 2 sex chromosomes. Autosomes are acrocentric, meaning that their centromeres are terminal. Liu, Y. *et al*.^1^ estimated *Bos taurus* genome size to be 2.87 Gb based on measurement from ESTs and previous genome size. Other studies referenced in Animal Genome Size Database^2^ based on flow cytometry, ultraviolet microscopy or biochemical analyzes have found genome sizes ranging from 3.15 Gb to 3.93 Gb. We estimated the genome size using k-mers with Jellyfish^3^ and Genomescope2.0^4^ on PacBio Sequel II HiFi data. Genomescope2.0 provided an estimate of 2.975 Mb with an heterozygosity of 0.281% and 36.9% of repeated sequences. The current ARS-UCD1.2 reference assembly metrics are: 2.715 Gb total size, 103.3 Mb N50 and 95.8% BUSCO score (See Table 2). We used ARS-UCD1.2 assembly as the reference to evaluate the quality of our assemblies.

### Sample collection & technical validation

Blood samples were collected, and stored with EDTA. The Wizard genomic DNA extraction kit (Promega) was used to extract frozen DNA from the three individuals. DNA was stored at −20 °C. Genomic DNA concentrations were measured using the Qubit fluorimetry system (Life Technologies) with the High Sensitivity(HS) kit for detection of double-stranded DNA (Thermo Fisher, Part #Q32854). Fragment size distributions were assessed using the Femto pulse Genomic DNA 165 kb Kit (Agilent, FP-1002-0275). Purity was measured using a Nanodrop system (Thermofisher). All samples were purified with AMPureXP beads (Beckman Coulter, A63882) to obtain correct ratios *i.e*. 260/280: 1.8–2 and 260/230:2-2.2. Degraded samples were re-extracted or sized using BluePippin Size Selection system (Sage Science) or circulomics kit.

### Sequencing

All sequencing processes were performed at the GeT-PlaGe core facility at INRAE Toulouse, 10.17180/NVXJ-5333.

#### Illumina paired-end WGS 2 × 250 bp

DNA-seq libraries were prepared according to the Illumina protocol TruSeq DNA PCR-Free High Throughput Library Prep Kit (96 samples, 20015963). DNA was fragmented by sonication on Covaris M220, size selection was performed using Sample Purification Beads of the library kit (ratio 1/1 beads water) and adaptators IDT for Illumina – TruSeq DNA UD Indexes (96 Indexes, 96 Samples, 20022370) were ligated before sequencing. Library quality was assessed using a Fragment Analyzer (Agilent)with High Sensitivity NGS Kit (DNF-474-0500). Sizes of 800 bp were obtained. Libraries were quantified by qPCR using the KAPA Library Quantification Kit (Roche, 07960140001). DNA was sequenced on two SP NovaSeq 6000 lanes using a paired-end read length of 2 × 250 bp with the Illumina SP Reagent kit (500 cycles). Both lanes produced respectively 118 Gb and 219 Gb, which corresponds to 112X coverage.

#### Hi-C

The Hi-C library was prepared according to a protocol described previously^5^. A sample of fresh blood was spun down, and cell pellet was resuspended and fixed in 1% formaldehyde. 5 million cells were processed for the Hi-C library. After overnight digestion with HindIII (NEB), DNA ends were labeled with Biotin-14-DCTP (Invitrogen) using the klenow (NEB) and religated. 1.4 microgramme of DNA was sheared to an average size of 550 bp (Covaris). A Qubit Fluorometer (HS kit) and a Fragment analyzer (NGS kit) were used to control DNA concentration and size. Biotinylated DNA fragments were pulled down using M280 Streptavidin Dynabeads (Invitrogen) and ligated to PE adaptors (Illumina). The Hi-C library was amplified using paired-end primers (Illumina) for 10 cycles. Sequencing was performed on on one S4 lane NovaSeq 6000 lanes using a paired-end read length of 2 × 150 bp. We produced around 25X coverage Hi-C data.

#### 10X genomics chromium technology

Linked reads libraries were prepared for the heifer and her parents according to 10XTM Genomics protocols using the Genome Library Kit & Gel Bead Kit v2, 16 rxns (PN-120258). Sample quantity and quality controls were validated on Qubit (HS kit), Nanodrop and Femto (DNA 165 kb Kit). Libraries were prepared from 3 µg of High Molecular Weight gDNA (cut off at 50 kb using BluePippin system, with “0.75% DF Marker U1 high pass 30–40 kb VS3” protocol, BUF7510). In the microfluidic Genome Chip, a library of Genome Gel Beads is combined with HMW template gDNA in Master Mix and partitioning oil to create Gel Bead-In-EMulsions (GEMs) in the Chromium. Each Gel Bead is functionalized with millions of copies of a 10X^TM^ Barcoded primer (i7 Multiplex Kit PN-120262). Upon dissolution of the Genome Gel Bead in the GEM, primers containing (i) an Illumina R1 sequence (Read 1 sequencing primer), (ii) a 16 bp 10x Barcode, and (iii) a 6 bp random primer sequence are released. Read 1 sequence and the 10X^TM^ Barcode are added to the molecules during the GEM incubation. P5 and P7 primers, Read 2, and Sample Index are added during library construction. 10 PCR cycles were applied to amplify libraries. Library quality was assessed using a Fragment Analyser (NGS kit) and libraries were quantified by qPCR using the Kapa Library Libraries were sequenced on a Illumina MiSeq with MiSeq Reagent Nano Kit v2 (300-cycles, MS-103-1001) to check equimolarity and quality then on a Illumina Novaseq 6000 S4 lane using a paired-end read length of 2 × 150 bp with the S4 Illumina Novaseq 6000 sequencing kits (300 cycles). Reads produced on the heifer and its parents were simultaneously sequenced of a S4 line, producing 801 Gb so on average 89 X par individual.

#### PacBio Sequel II – CCS (for HiFi reads) and CLR Libraries

At each step, DNA was quantified using the Qubit HS kit. DNA purity was tested using a Nanodrop and size distribution and degradation assessed using the Femto pulse DNA 165 kb Kit. Purification steps were performed using AMPure PB beads (PacBio 100-265-900).

Library preparation was performed according to the manufacturer’s instructions “Procedure & Checklist Preparing HiFi SMRTbell Libraries using SMRTbell Express Template Prep Kit 2.0”(100-938-900).

For CLR library: 30 µg of DNA was purified then sheared at 60 kb using the Megaruptor3 system (Diagenode). Using SMRTbell Express Template prep kit 2.0, a Single strand overhangs removal, a DNA and END damage repair step were performed on 10 µg of sample. Then blunt hairpin adapters were ligated to the library. A size selection step using a 30 kb cutoff was performed on the BluePippin Size Selection system with “0.75% DF Marker U1 high pass 30–40 kb VS3” protocol. A 70 kb library was recovered. Using Binding kit 2.0 kit (101-789-500) and sequencing kit 2.0 (101-820-200) with 1 h of annealing and 4 h of binding, the primer V4 annealed and polymerase 2.0 bounded library was sequenced by diffusion loading onto 2 SMRTcells 8 M (101-389-001) on Sequel II instrument at 50 to 70 pM with a 15 hours movie. A coverage of 42X was obtained.

For HiFi library: 30 µg of DNA was purified then sheared at 15 kb using the Megaruptor3 system (Diagenode, N°E07010003). Using SMRTbell Express Template prep kit 2.0, a Single strand overhangs removal, a DNA and END damage repair step were performed on 10 µg of sample. Then blunt hairpin adapters were ligated to the library. The library was treated with an exonuclease cocktail to digest unligated DNA fragments. A size selection step using a 12 kb cutoff was performed on the BluePippin Size Selection system with “0.75% DF Marker S1 3–10 kb Improved Recovery” protocol (BLF7510). The first fraction was discarded, the second was recovered in manual mode to obtain a 20 kb library. Using Binding kit 2.0 kit and sequencing kit 2.0 with 1 h for annealing and 1 h for binding, the primer V2 (101-847-900) annealed and polymerase 2.0 bounded library was sequenced by diffusion loading onto 6 SMRTcells on Sequel II instrument at 50 to 70 pM with a 2 hours pre-extension and a 30 hours movie. A coverage of 40X HiFi was obtained.

#### ONT

At each step, DNA was quantified using the Qubit HS kit. DNA purity was tested using the nanodrop and size distribution and degradation assessed using the Fragment analyzer (AATI) High Sensitivity DNA Fragment Analysis Kit. Purification steps were performed using AMPure XP beads (Beckman Coulter). Library preparation was performed according to the manufacturer’s instructions “1D gDNA selecting for long reads (SQK-LSK109)”. For 1 Flowcell, 5 µg of DNA was purified then sheared at 20 kb using the Megaruptor system (Diagenode). A 10 kb size selection step using Short Read Eliminator XS Kit (circulomics) or using the BluePippin Size Selection system was performed. A one-step DNA damage repair + END-repair + dA tail (NEB) of double stranded DNA fragments was performed on 1 µg of sample. Then adapters were ligated to the library. The library was loaded onto three FLO-MIN106D and two FLO-PRO002 (R9.4.1) flowcells and sequenced on GridION and PromethION instruments at 20 pmol within 72 h. Nuclease flush steps were done when necessary and possible, i.e. when only 30% of the pores were still in sequencing. We produced 52X.

### Genome assemblies

**Fig. 2:**
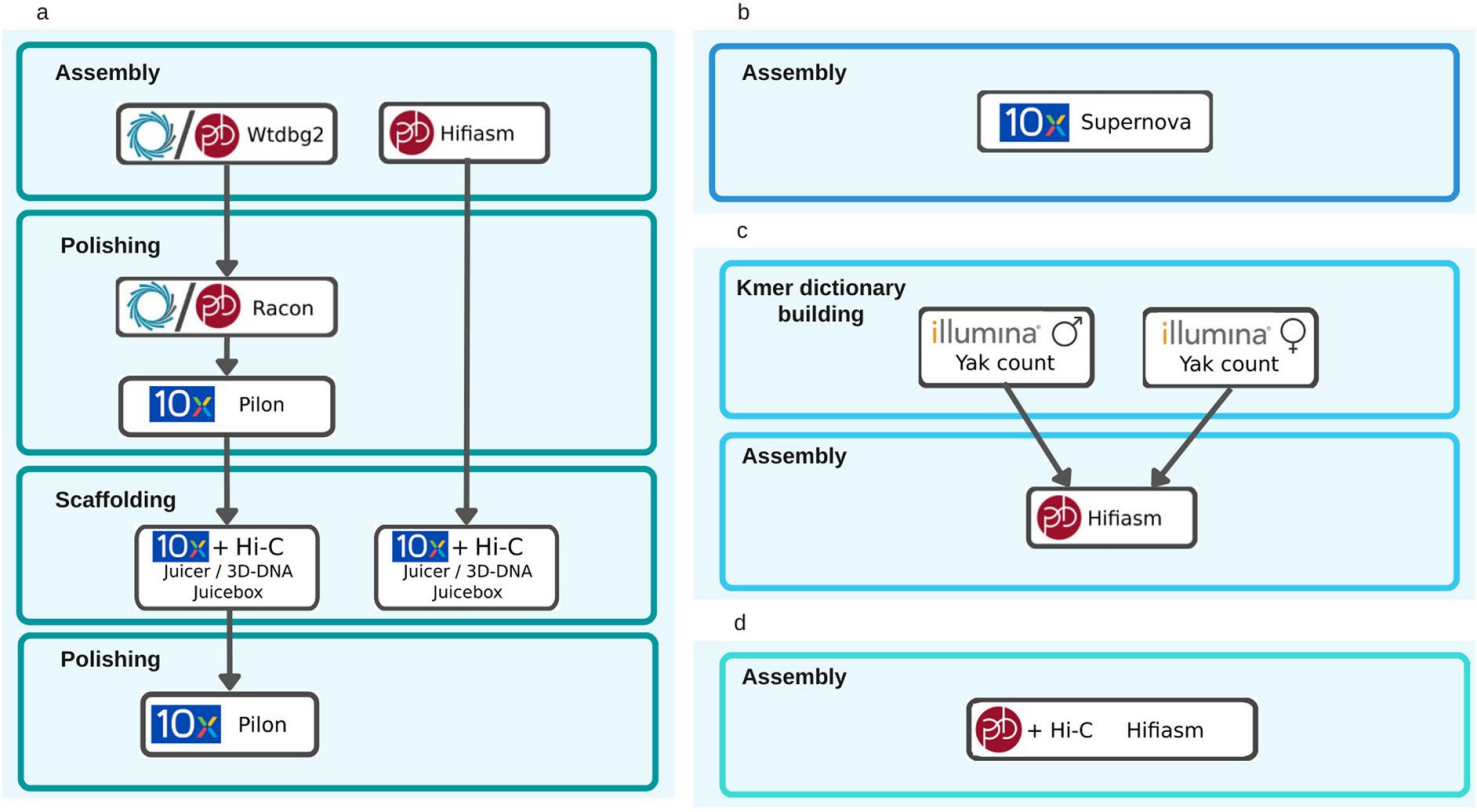
Details of the 5 pipelines used to produce our assemblies. a-Long reads assemblies from Oxford Nanopore Technologies and Pacific Biosciences followed by polishing step for erroneous assemblies and scaffolding step. b-10X Chromium assembly and scaffolding with Supernova. c-Phased assembly with HiFi and parental illumina reads. d-Phased assembly with HiFi and and Hi-C data.

To evaluate the contribution of the different technologies to the genome assembly, we generated one assembly of the heifer for each data type (See Fig. 2 for detailed pipelines). First, we assembled Oxford Nanopore reads with wtdbg2. Read coverage for this assembly was 52X (See Table 1). To improve assembly sequence quality, we polished it with Racon^6^ and Pilon^7^. For this, ONT reads were aligned with minimap2^8^ and processed with Racon using default parameters. Then, the 89X 10X Chromium reads were aligned on the resulting contigs with longranger^9^ and the alignment file was processed with pilon using default parameters. In addition, the previously used 10X Chromium reads were assembled with Supernova^10^. We also assembled PacBio CLR reads with wtdbg2 using 42X coverage. Finally, 40X PacBio HiFi reads from heifer were assembled using hifiasm^11^. Because hifiasm is able to mix different data types to produce phased assemblies, we performed three HiFi assemblies for the heifer. The first assembly is a traditional consensus assembly, while the other two are phased assemblies meaning that both of the chromosomal copies are assembled separately (one for each parent). The phasing quality and phased assembly metrics are analyzed in detail in the Phasing section. To check Hi-C reads quality, we scaffolded our assemblies using Juicer^12^ and 3D-DNA^13^ and then compared the result to the bovine reference genome ARS-UCD1.2. To do this, we first scaffolded the assemblies with Hi-C reads and manually corrected the resulting contact map with Juicebox^14^ (See Fig. 8a). The contact map contigs organisation shown by juicebox is considered correct if the contact density is maximal on the diagonal. Chromosomes should also be clearly separated in the map by areas with a very low contact density. Both criteria were visualy inspected. When these criteria were not fullfilled contigs were moved, reversed or split in order to maximize contact signal on the diagonal and chromosome separation. Finally, we loaded a contact map generated from 10X linked reads presenting very local contact information in Juicebox in order to visually check the scaffold organization (See Code availability).

**Table 1:** Summary of data used in this study. Characteristics of these data for the Charolais trio are described here, and in more detail in this manuscript. For information about pipeline used in this study, refer to Fig. 2.

Sample	Sequencing platform	Instrument/mode	Quantity	Insert size (bp)	Read length (bp)	Number of Gbases	Sequence depth X	ID	Pipeline use
Heifer	Oxford Nanopore	GridION/ PromethION	3 flowcells GridIONs - 2 flowcells PromethION	NA	25000 to 45000	154	52	ERR10386215 to ERR10386219	a
Heifer	Illumina	NovaSeq	2 lanes S4	800	2 × 250 bp PCR Free	337	112	ERR10310239, ERR10310240	a, c
Heifer	PacBio	Sequel II	1 smrtcells CLR	NA	100000	278	42	ERR10378053	a
Heifer	PacBio	Sequel II	5 smrtcells CCS	NA	15000 to 20000	119	40	ERR10378054 to ERR10378058	c, d
Heifer	Hi-C	NovaSeq/HiSeq 3000	1 lane SP + 1 lane HiSeq	434	2 × 150 bp	75/sample	25/sample	ERR10310241, ERR10310244	a, c
Trio	Chromium 10X	NovaSeq /MiSeq	1 lane S4/ 1 run MiSeq	500	2 × 150 bp	267/sample	89/sample	heifer: ERR10310247 ERR10310250 father: ERR10310248 ERR10310251 mother: ERR10310249 ERR10310252	a, b, c

### Structural variation

To evaluate the contribution of the different technologies for structural variant (SV) detection, a structural variant analysis was performed for each of the available datasets. By structural variation we mean the differences between the Charolais heifer genome and the ARS-UCD 1.2 bovine reference assembly. The general approach used was to map the reads from all technologies to the ARS-UCD 1.2 bovine reference assembly, followed by an SV detection using state-of-the-art detection software for each technology. More specifically, for the ONT data set, the reads were aligned using minimap2^8^ with sequencing technology specific parameters (see below), and SV detection was performed using SVIM^15^. For PacBio CLR and HiFi, reads were aligned using pbmm2^16^ and SV detection was performed using pbsv^17^. For the Illumina dataset, the reads were aligned using bwa mem^18^ and SVs were detected using the manta software^19^. We also took advantage of the assembly produced for the heifer using PacBio Sequel II HiFi reads (see Assembly production). For the phased assembly, the two corresponding haplotypes were aligned to the ARS-UCD1.2 bovine assembly using minimap2^8^ and SVs were detected using SVIM-ASM^20^. The SV sets of the different technologies were merged using Jasmine^21^ which enabled the identification of the shared SVs and the technology-specific SVs.

## Data Records

All read files have been uploaded to ENA European Nucleotide Archive^22^ and can be accessed on^23^. Table 2 contains the run accession numbers per sample for the runs used to produce our seqoccin.Bt.char.v1.0 assembly and both our Trio phased assemblies. Our seqoccin.Bt.char.v1.0 assembly is also available on^24^.

**Table 2:** Summary of heifer produced contigs assemblies. For details about pipeline used in this study, refer to Fig. 2. *BUSCO analysis was performed on polished contigs, **Inspector Quality Value is calculated on reference alignment andreads alignment.

	ARS-UCD1.2	ONT wtdbg2	10X supernova	HiFi hifiasm	CLR wtdbg2
Pipeline used		a	b	a	a
Data type	CLR	ONT	10X Chromium	CCS	CLR
Quantity	80X	58X	95X	40X	43X
Assembler	Falcon	Wtdbg2	Supernova	Hifiasm	Wtdbg2
Number of contigs	3 077	7 226	26 306	1 444	2 857
Total size	2 700 000 000	2 701 288 401	2 627 892 463	3 244 632 679	2 631 921 359
N50 contigs length	12 000 000	23 641 545	488 571	84 059 894	16 542 341
BUSCO	95.7%*	C:70.2%	C:94.7%	C:95.9%	C:90.0%
Inspector QV **		22.29	27.09	47.25	25.79

The others assembly files in fasta format can be accessed on^25^ and^26^ for the phased assemblies. All the structural variation files in vcf format can be accessed on^27^.

## Technical Validation

Read sets technical validation was performed first by producing read metrics, second by assessing read assembly quality and last by checking the correspondence of the variants found using each technology. Illumina data were basecalled with bcl2fastq2.10, and PacBio data with the current version of SMRTLink at the time of sequencing. For ONT fastq data, the basecaller version is indicated in the metadata.

### Raw read quality

**Fig. 3:**
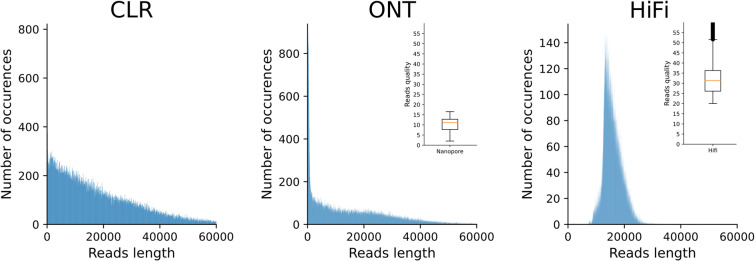
Plot of the length and quality of our long reads datasets. The main graph of each technologies shows the histogram of size distribution from the randomly chosen 100 000 reads, in the corner of this graph is a boxplot of the quality of theses reads. Metrics are based on fastq file output and are technology dependent. CLR quality is not computed during fastq production and therefore not exploited. HiFi quality is not phred-score based.

A random selection of 100,000 reads for each set was used to produce a per read mean nucleotide quality and read length distribution plot with seqkit^28^ (See Fig. 3). All the values are in agreement with the libraries produced, thus showing the good quality of the data used.

Mapping the reads to the ARS-UCD1.2 reference assembly resulted in 119X coverage and more than 99% of genome covered by at least 50 reads for Illumina, 54X and more than 99% of genome covered by at least 10 reads for ONT, 42X and more than 99% of genome covered by at least 10 reads for HiFi, and 34X and more than 99% of genome covered by at least 10 reads for CLR.

In addition, Juicer^12^ was used to align Hi-C reads on the ONT wtdbg2 polished assembly. From around 250 million read pairs sequenced, more than 94% are considered as alignable (Normal paired + Chimeric paired). More than 72% are unique and for the final Hi-C map elaboration, we obtained 151,865,989 pairs which represent 15X useful coverage.

### Assembly completeness

**Fig. 4:**
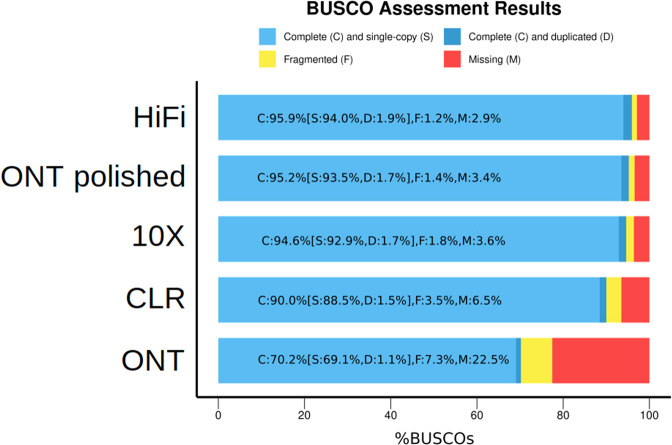
BUSCO plot of different Heifer assemblies. Summary plot produced with BUSCO V5.1 and mammalia odb10 dataset.

**Fig. 5:**
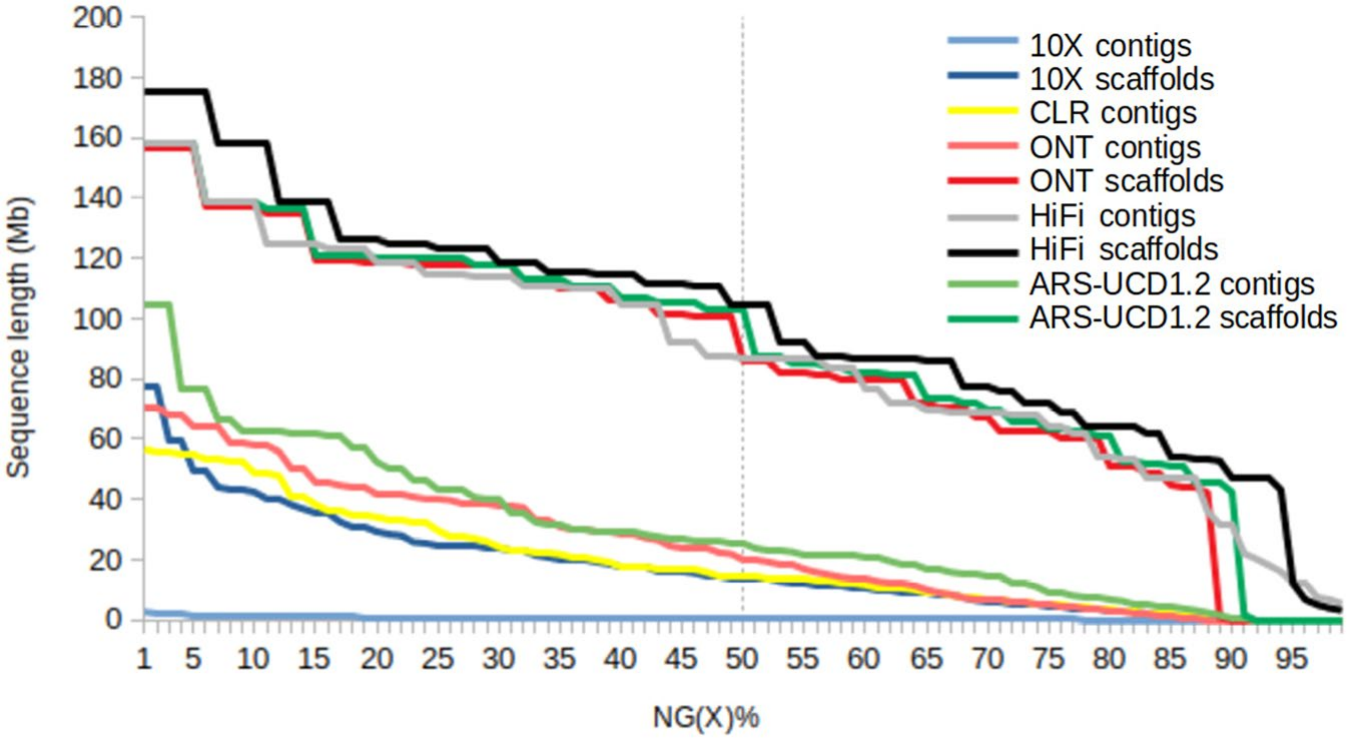
Heifer NG(X)% comparison. Comparison and evolution of the heifer assemblies size metrics after different steps in different assemblies pipelines.

To check the potential of each data type to produce an accurate and complete assembly, we evaluated the completeness of our bovine assemblies (10X supernova, ONT wtdbg2, CLR wtdbg2, HiFi hifiasm) and compared their metrics with *Bos taurus* reference ARS-UCD1.2 (See Figs. 4, 5 and Table 2 for detailed information). The assemblies sizes were close to the 3.0 Gb Genomescope estimation, ranging from 2.6 Gb for 10X chromium, 2.7 Gb for ONT and CLR wtdbg2 assemblies to 3.2 Gb for HiFi hifiasm assembly. These values represent between 86.6% and 106.6% of the expected genome size. The HiFi hifiasm assembly is slightly longer than other assemblies, since the high read quality makes it possible to distinguish different copies of repeated regions, found, for instance, in centromeres and telomeres (see Fig. 8 for detailed information about repeated areas). Thanks to this information, Hifiasm can correctly assemble areas previously missing or mis-assembled by other assemblers or read types. The assembly size difference was annotated by repeatModeler^29^ as 500 Mb of complex repeats (see Fig. 8). In terms of contig sizes, the HiFi hifiasm assembly had the largest contig N50 of 84 Mb, followed by ONT wtdbg2 and CLR wtdbg2 having 23 Mb and 16.6 Mb respectively. Since Supernova assembler produces a scaffolded assembly (using the long-range information from 10X linked reads), we present both contig and scaffold metrics. Contigs have N50s of 489 kb, while corresponding scaffolds have 15 Mb N50s.

The assemblies were also subjected to Benchmarking Universal Single-Copy Orthologs^30^ which evaluates genome completeness using proteins expected in unique copy. Mammalia release 10 dataset (mammalia.odb10) was used here. Assemblies made from reads with low error rates, such as HiFi hifiasm and 10X supernova assemblies had, as expected, a much higher BUSCO score (>90% complete sequences). Assemblies produced from long error-rich reads as CLR or ONT have a lower BUSCO score (< = 90%), mainly because read sequencing errors had poorly been corrected in the consensus. Assemblies were compared using inspector^31^, which produces metrics including the number of misassemblies, the duplication ratio and the k-mer-based completeness. Inspector metrics in Table 2 shows assembly quality values (QV) ranging from 22.29 for the ONT wtdbg2 to 47.25 for the Hifi hifiasm. The alignment of these assemblies against the reference gave a coverage greater than 94.2% and read alignment against the assemblies gave alignment rates greater than 99.75%. These values attest a good read production, allowing to produce high quality assemblies. The best assembly being the one produced hifiasm using HiFi reads.

### Assembled sequence accuracy

Assemblies produced from reads with high error rate require polishing in order to increase their consensus quality. We polished ONT wtdbg2, firstly using the same ONT reads with Racon and then using 10x Chromium reads with Pilon. Both polishing steps slightly changed the assembly metrics (<0.1% change in Total size, N50 and longest contigs) but greatly improved the BUSCO score, with + 10% increase after Racon polishing and around 15% more after Pilon polishing (see Table 3). After both steps, the assembly BUSCO score increased from 70.2% to 95.2% which represents an increase of 25%. At the end of these polishing steps, less than 5% of expected genes are still missing, which represents 317 of the 9226 genes evaluated by BUSCO. With a BUSCO scores above 95% and few errors in the reads, HiFi and 10X assemblies do not require polishing.

**Table 3:** Summary of heifer polished assemblies. Only ONT contig assembly was polished as CCS and 10X are low error rate reads. As CLR assembly is better than ONT assembly, we can expect at least similar result after polishing step. For details about pipeline used in this study, refer to Fig. 2.

	ARS-UCD1.2	ONT wtdbg2 polished
Pipeline used		a
Data type	CLR/Illumina	ONT/10X
Quantity	80X/83X	58X/95X
Polisher	Quiver + Arrow + Pilon	Racon + Pilon
Number of contigs	2 596	5 783
Total size	2 715 825 655	2 700 580 867
N50 contigs length	25 896 116	23 984 524
BUSCO	C:95.7%	C:95.2%

### Chromosome scaffolding

After the Juicer^12^ - 3D-DNA^13^ - Juicebox^14^ scaffolding pipeline, 97% of the contigs were placed in scaffolds. The 30 largest scaffolds represent 95.3% of the full assembly. Final BUSCO metrics showed a high completeness of all 3 finished assemblies, ranging from 94.7% to 95.8% of found complete genes, with around 1.3% of fragmented genes (See Table 4). To check scaffolding quality, the assemblies were aligned against the reference to produce dot-plots with DGenies^32^ (see Fig. 8). These alignments showed concordance between scaffolds and reference chromosomes over the entire genome, with the exception of a few small areas, representing possible intra-contig reorganizations.

**Table 4:** Summary of heifer produced chromosomes assemblies. As CLR assembly is better than ONT assembly, we can expect at least similar result after polishing and final steps. For details about pipeline used in this study, refer to Fig. 2.

	ARS-UCD1.2	ONT wtdbg2 polished	10X supernova	HiFi hifiasm
Pipeline used		a	b	a
Data type	Hi-C/Optical + Recombination map	Hi-C/10X	10X Chromium	Hi-C/10X
Quantity	84X Hi-C	28X/95X	95X	28X/95X
Scaffolder	HiRise + IrysView	3D-DNA + Juicer + Juicebox	Supernova	3D-DNA + Juicer + Juicebox
Number of scaffolds	2 211	4 600	15 291	1 391
Total size	2 715 853 794	2 705 347 253	2 663 443 665	3 244 660 179
N50 scaffolds length	103 308 737	100 959 810	15 206 899	87 697 707
BUSCO	C:95.8%	C:95.2%	C:94.7%	C:95.8%
Inspector QV		32.34	27.09	47.76

In terms of produced chromosomes metrics, our ONT wtdbg2 assembly is close to the ref. ^30^: chromosomes, around 2.7 Gb total size, 156 Mb for the longest chromosome and a 101 Mb of N50. The HiFi hifiasm assembly is around 17% larger than the reference assembly, mainly due to additional repeated sequences assembled, but shares close metrics.

### Phasing

Hifiasm produces phased assemblies and can take advantage of Hi-C reads information as well as trio k-mers (see Fig. 2c,d). First, HiFi reads (40X) were combined with Hi-C reads from the same individual (25X), for an assembly that we called HiFi hifiasm Hi-C. The HiFi reads were then combined with parental k-mer dictionary extracted from 10X chromium reads (89X), for an assembly that we called HiFi hifiasm parent. Both HiFi hifiasm Hi-C and parent assemblies were larger than the reference, their total size ranging from 3.08 Gb to 3.18 Gb with an N50 around 70 Mb, and a BUSCO score greater than 95% in both cases (See Table 5). Haplotype quality was checked. In HiFi hifiasm Hi-C assemblies, haplotype2 was 100 Mb larger than haplotype1, mainly due to misphased contigs creating duplication in haplotype2. Haplotype2 also had 9 Mb larger N50 but BUSCO scores were similar between both haplotypes (95.7% and 95.8%). For HiFi hifiasm parent assemblies, both haplotypes metrics were close (3.16 Gb vs 3.11 Gb and 71 Mb vs 69 Mb N50), but BUSCO scores were slightly different (95.8% for haplotype1 and 95.3% for haplotype2). Having no phased reference to evaluate these assemblies, we tested an in-house protocol (see code availability section) to estimate phasing quality as the proportion of misplaced haplotypes in each assembly. First, k-mer dictionaries was created for each assembly (HiFi hifiasm parent hap1 and hap2) and each origin (paternal reads, maternal reads). Ambiguous and homozygous k-mers were discarded: all the k-mers with low coverage in reads and those present in both paternal and maternal reads or in both haplotype1 and haplotype2 assemblies. assembly and read k-mer dictionaries were then pairwise compared. A phasing value of each assembly was then computed corresponding to $\frac{{n}_{{\rm{maj}}}}{{n}_{{\rm{maj}}}+{n}_{{\rm{\min }}}}\times 100$, where *n*_maj_ and *n*_min_ is the number of paternal k-mers in the supposed paternal haplotype (majority) and the number of maternal k-mer (minority), and vice-versa for the other haplotype. Haplotype separations of 97.3% and 96.7% for HiFi hifiasm parent assemblies and 62.6%, 60.5% for HiFi hifiasm Hi-C assemblies were obtained. The lower HiFi hifiasm Hi-C assembly phasing quality comes from the fact that Hi-C data is not optimal to separate contigs with distinct parental origins. Therefore the same calculation was performed on the contig level, which produced intra-contig separations are 84.6% and 85.6% showing that the Hi-C separation is less efficient than parental separation, but works fine on a contig level even if parental data is not available. To conclude, contig haplotyping worked with both approaches, HiFi hifiasm parent method being more efficient than HiFi hifiasm Hi-C. HiFi hifiasm Hi-C required additional processing, such as manual reorganization of the contigs in the haplotypes, in order to obtain good quality assemblies.

**Table 5:** Summary of heifer phased produced assemblies. For details about pipeline used in this study, refer to Fig. 2. *BUSCO analysis was performed on polished contigs. **Reference is not haplotyped.

	ARS-UCD1.2	HiFi hifiasm parent hap1	HiFi hifiasm parent hap2	HiFi hifiasm Hi-C hap1	HiFi hifiasm Hi-C hap2
Pipeline used		c	c	d	d
Data type	CLR	CCS + trio	CCS + trio	CCS + Hi-C	CCS + Hi-C
Quantity	80X	40X	40X	40X + 28X	40X + 28X
Assembler	Falcon	Hifiasm	Hifiasm	Hifiasm	Hifiasm
Number of contigs	3 077	2 871	2 300	2 658	2 136
Total size	2 700 000 000	3 156 028 877	3 113 483 345	3 077 978 241	3 184 033 110
N50 contigs length	12 000 000	71 619 842	69 165 538	80 106 842	71 644 334
BUSCO	95.7%*	95.8%	95.3%	95.8%	95.7%
Phasing ratio	**	97.3%	96.7%	62.6%	60.5%
Contigs phasing ratio	**	97.5%	96.9%	84.6%	85.6%

#### Structural variation

**Fig. 6:**
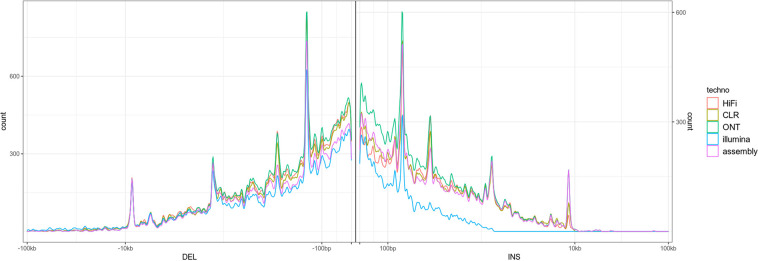
Length distribution of deletions (left) and insertions (right), detected by the different technologies on the Charolais heifer (see Methods). The different peaks at 100 bp, 200 bp, 5 kb and 10 kb correspond to structural variations due to transposable elements families (SINE, LTR, LINE). These distributions should be by construction symmetric because the comparison between the Charolais heifer genome and the Dominette genome of the ARS-UCD1.2 reference assembly is by essence symmetric. A departure from symmetry is an indication of a biais. The Illumina technology is clearly biased towards deletions due to the difficulty to recover medium to long insertions with short reads, this biais stands although for short insertions. In contrast, long read technologies exhibit no clear biais suggesting that they are able to correctly detect insertions and deletions in this large spectra. The Bionano technology exhibits a very different pattern with a difficulty to identify small variants, a behavior that was expected.

**Fig. 7:**
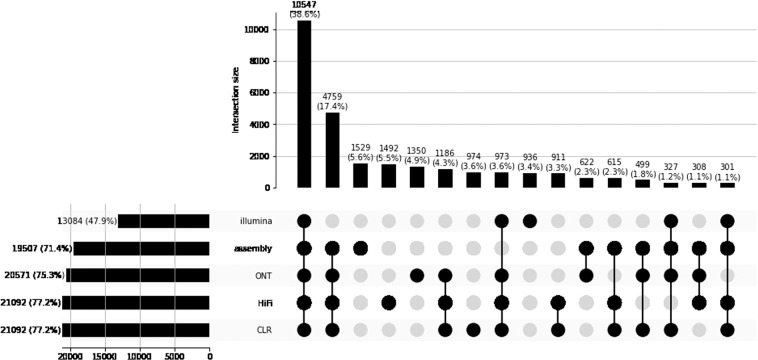
Upset plot of the identified deletions and insertions. Most of the variants are identified by all the long read technologies as well as for the assembly comparison based variant detection. This Upset plot underlines again the added value of long reads for variant detection.

The sequencing data made available here allows first to comprehensively analyze the structural variability for a typical bovine, and second to benchmark the different sequencing technologies and associated detection software for structural variation discovery. The size distribution of the structural variants detected on these datasets (Fig. 6) illustrates these two points. First, it illustrates the specific bovine structural variability with peaks corresponding to SINEs, LTRs, and LINEs. Second, it illustrates the behavior of the different technologies with respect to variant detection, such as the size spectrum of the different technologies. For example, the obvious size limitation for insertion detection using Illumina data. A large proportion of the variants were shared by the different long-read technologies SV set, and also by the assembly-based structural variation discovery (Fig. 7).

**Fig. 8:**
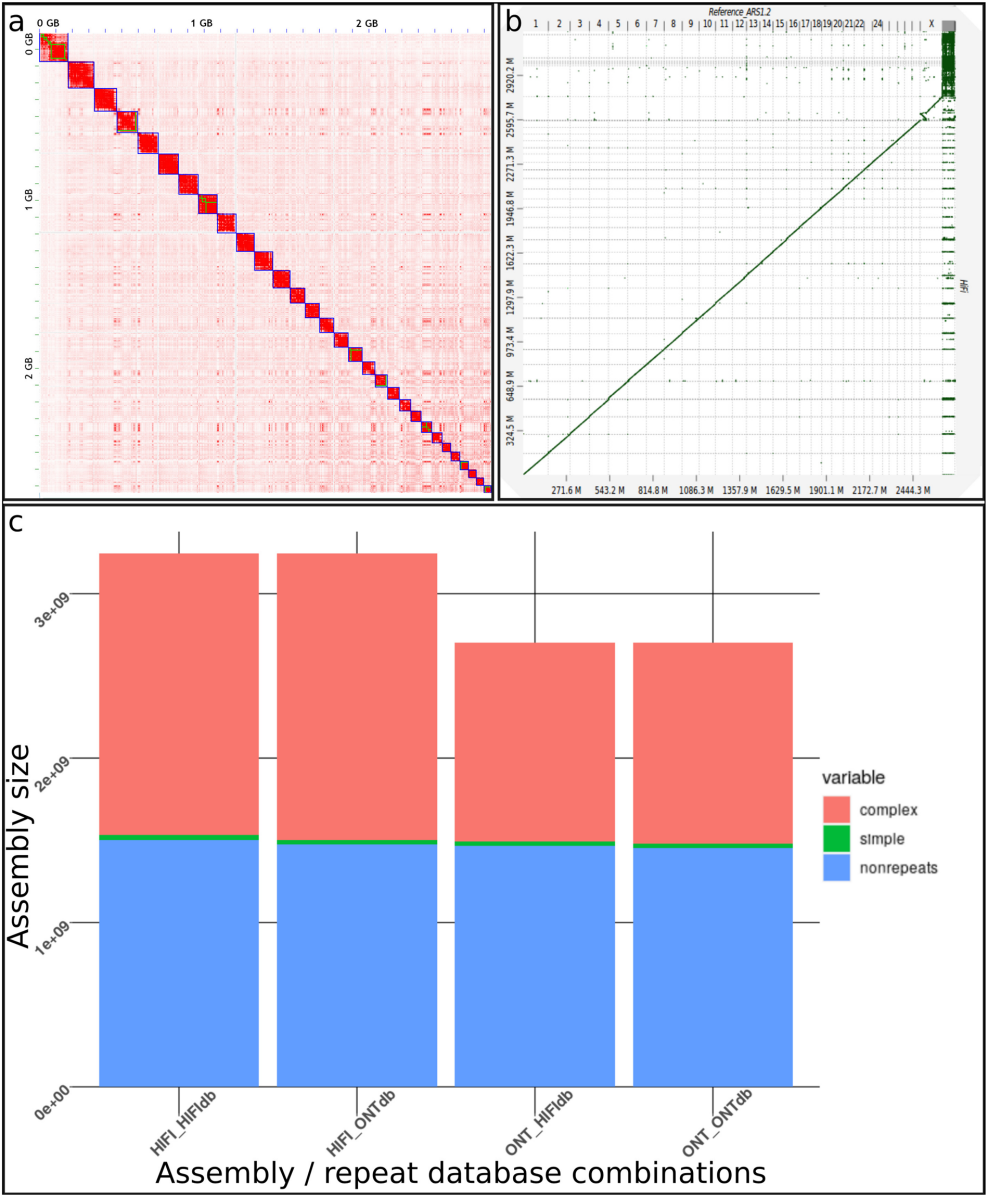
a-Hifi assembly from heifer Hi-C contact matrix visualization with Juicebox. Green squares represent original contigs, blue squares the manually produced scaffolds and each red dot an Hi-C contact. b-Dgenies Dot-Plot of HiFi Final assembly against bos taurus reference ARS-UCD1.2. Scaffolded assembly (Y-axis) were aligned to ARS-UCD1.2 chromosomes (X-axis) using minimap2 c-RepeatMasker/RepeatModeler representation of HiFi assembly and ONT assembly. The additionnal information in HiFi assembly is mainly Complex duplications.

## Data Availability

The scripts enabling to reproduce the assemblies presented in this manuscript are available on a dedicated Web page: [https://github.com/GeTPlaGe/SeqOccIn/tree/main/Data%20paper/Bos%20taurus%20data%20paper].

## References

[1] Liu, Y. *et al*. Bos taurus genome assembly. *BMC Genomics* **10**, 10.1186/1471-2164-10-180 (2009). 10.1186/1471-2164-10-180 PMC2686734 19393050

[2] Gregory, T. R. Animal genome size database. http://genomesize.com (2023).

[3] Marçais G, Kingsford C (2011). A fast, lock-free approach for efficient parallel counting of occurrences of k-mers. Bioinformatics.

[4] Vurture GW (2017). GenomeScope: fast reference-free genome profiling from short reads. Bioinformatics.

[5] Foissac S (2018). Transcriptome and chromatin structure annotation of liver, CD4+ and CD8+ T cells from four livestock species.

[6] Vaser R, Sović I, Nagarajan N, Šikić M (2017). Fast and accurate de novo genome assembly from long uncorrected reads. Genome Research.

[7] Walker BJ (2014). Pilon: An integrated tool for comprehensive microbial variant detection and genome assembly improvement. PLoS ONE.

[8] Li H (2018). Minimap2: pairwise alignment for nucleotide sequences. Bioinformatics.

[9] Zheng GXY (2016). Haplotyping germline and cancer genomes with high-throughput linked-read sequencing. Nature Biotechnology.

[10] Mostovoy Y (2016). A hybrid approach for de novo human genome sequence assembly and phasing. Nature Methods.

[11] Cheng H, Concepcion GT, Feng X, Zhang H, Li H (2021). Haplotype-resolved de novo assembly using phased assembly graphs with hifiasm. Nature Methods.

[12] Durand NC (2016). Juicer provides a one-click system for analyzing loop-resolution hi-c experiments. Cell systems.

[13] Dudchenko O (2017). De novo assembly of the *Aedes aegypti* genome using Hi-C yields chromosome-length scaffolds. Science.

[14] Durand NC (2016). Juicebox provides a visualization system for hi-c contact maps with unlimited zoom. Cell Systems.

[15] Heller D, Vingron M (2019). SVIM: structural variant identification using mapped long reads. Bioinformatics.

[16] Pacific BioSciences. *A minimap2 SMRT wrapper for PacBio data*. https://github.com/PacificBiosciences/pbmm2.

[17] Pacific BioSciences. *PacBio structural variant calling and analysis tools*. https://github.com/PacificBiosciences/pbsv.

[18] Li H (2013). Aligning sequence reads, clone sequences and assembly contigs with bwa-mem.

[19] Chen X (2015). Manta: rapid detection of structural variants and indels for germline and cancer sequencing applications. Bioinformatics.

[20] Heller D, Vingron M (2020). SVIM-asm: structural variant detection from haploid and diploid genome assemblies. Bioinformatics.

[21] Kirsche, M. *et al*. Jasmine: Population-scale structural variant comparison and analysis. *bioRxiv* https://www.biorxiv.org/content/early/2021/05/28/2021.05.27.445886.full.pdf, 10.1101/2021.05.27.445886 (2021).

[22] Leinonen R (2010). The European nucleotide archive. Nucleic acids research.

[23] (2022). European Nucleotide Archive.

[24] Eche C (2022). GenBank.

[25] Eche C (2022). Recherche Data Gouv.

[26] Eche C (2022). Recherche Data Gouv.

[27] Eche C (2022). Recherche Data Gouv.

[28] Shen W, Le S, Li Y, Hu F (2016). SeqKit: a cross-platform and ultrafast toolkit for FASTA/Q file manipulation. PloS ONE.

[29] Smit, A. & Hubley, R. *Repeatmodeler open-1.0* http://www.repeatmasker.org (2008).

[30] Manni M, Berkeley MR, Seppey M, Simão FA, Zdobnov EM (2021). BUSCO update: Novel and streamlined workflows along with broader and deeper phylogenetic coverage for scoring of eukaryotic, prokaryotic, and viral genomes. Molecular Biology and Evolution.

[31] Chen, Y., Zhang, Y., Wang, A. Y., Gao, M. & Chong, Z. Accurate long-read de novo assembly evaluation with inspector. *Genome Biology* **22**, 10.1186/s13059-021-02527-4 (2021). 10.1186/s13059-021-02527-4 PMC8590762 34775997

[32] Cabanettes F, Klopp C (2018). D-GENIES: dot plot large genomes in an interactive, efficient and simple way. PeerJ.

